# A Crank–Nicolson collocation spectral method for the two-dimensional telegraph equations

**DOI:** 10.1186/s13660-018-1728-5

**Published:** 2018-06-19

**Authors:** Yanjie Zhou, Zhendong Luo

**Affiliations:** 1 School of Science, Beijing Technology and Business University, Beijing, China; 2 School of Mathematics and Physics, North China Electric Power University, Beijing, China

**Keywords:** 65N30, 65N12, 65M15, Crank–Nicolson collocation spectral method, Telegraph equation, Existence, stability, and convergence, Numerical experiment

## Abstract

In this paper, we mainly focus to study the Crank–Nicolson collocation spectral method for two-dimensional (2D) telegraph equations. For this purpose, we first establish a Crank–Nicolson collocation spectral model based on the Chebyshev polynomials for the 2D telegraph equations. We then discuss the existence, uniqueness, stability, and convergence of the Crank–Nicolson collocation spectral numerical solutions. Finally, we use two sets of numerical examples to verify the validity of theoretical analysis. This implies that the Crank–Nicolson collocation spectral model is very effective for solving the 2D telegraph equations.

## Introduction

Because any bounded closed domain Ω̅ in $\mathbb{R}^{2}$ can be approximately filled with several rectangles $[a_{i},b_{i}] \times[c_{i},d_{i}]$ ($i=1, 2, \ldots, I$), for convenience and without losing universality, we just assume that $\overline{\Omega}=[a, b] \times[c, d]\subset \mathbb{R}^{2}$ with boundary *∂*Ω and consider the following two-dimensional (2D) telegraph equations:

1 $$\begin{aligned} \textstyle\begin{cases} u_{tt}-{\mu}\triangle u+\alpha u_{t}+\beta u=f(x,y,t), &(x,y,t)\in\Omega\times[0,T], \\ u(x,y,t)=\varphi(x,y,t), &(x,y,t)\in\partial\Omega\times[0,T], \\ u(x,y,0)=H(x,y),\qquad u_{t}(x,y,0)=G(x,y),&(x,y)\in\Omega, \end{cases}\displaystyle \end{aligned}$$

where *u* is the unknown function, $u_{tt}=\partial^{2}u/\partial t ^{2}$, $\triangle=\partial^{2}/\partial x^{2}+\partial^{2}/\partial y^{2}$ is the Laplace operator, *T* is the final time, $f(x,y,t)$, $\varphi(x,y,t)$, $H(x,y)$, and $G(x,y)$ are four given functions, and ${\mu}=(L\hat{C})^{-1}$, $\alpha=GR{\mu}$, and $\beta=(R\hat{C}+GL) {\mu}$ are three known positive constants because *G* is the conductance of the dielectric material, *R* is the distributed resistance of the conductor, *L* is the distributed inductance, and *Ĉ* is the capacitance between the two conductors. For convenience, but without losing generality, we further assume that $\varphi(x,y,t) = 0$.

The telegraph equations have a very significant physical background, so that they have become a type of important evolution partial differential equations (PDEs) and have been successfully used in many numerical simulations in mathematical and physical problems used to describe the propagation of an electric signal in a cable of transmission line and wave phenomena. Especially, they can be suitable for modeling the interaction between reaction and diffusion in physics and biology (see [1, 2]). Therefore, the study for the telegraph equations has significant meaning. However, the telegraph equations in the real-world problems usually include the complex known data, such as the complicated initial and boundary value conditions, the intricate source term, the discontinuous coefficients, so that they have no analytic solution. Thus we have to rely on numerical solutions.

The finite difference scheme (FDS), the finite element method (FEM), the finite volume element method (FVEM), and the spectral method are regarded to be four most popular methods, but the accuracy of the spectral method is highest in the four numerical methods because it adopts smooth functions (such as trigonometric functions, Chebyshev’s polynomials, Jacobi’s polynomials, and Legendre’s polynomials) to approximate unknown function, whereas FEM and FVEM usually adopt standard polynomials to approximate an unknown function, and FDS adopts difference quotient to approximate derivative. Particularly, with rapid development of computers, the spectral method has achieved great success in many numerical computing fields (see, e.g., [3, 4]). The spectral method is a weighted residual way for PDEs and generally is classified as the Galerkin spectral method, the spectral tau method, and the collocation spectral (CS) method, which are used to solve many PDEs including the second-order elliptic equations, parabolic equations, hyperbolic equations, and hydromechanics equations (see, e.g., [3–9]).

Although FDS, FEM, and FVEM have been used to solve the telegraph equations (see [1, 2, 10–16]), as far as we know, the spectral method, especially the CS method, has yet not been used to solve the 2D telegraph equations. Therefore, in this paper, we first develop a Crank–Nicolson CS (CNCS) model for the 2D telegraph equations. Then we analyze the existence, uniqueness, stability, and convergence for the CNCS solutions. Finally, we utilize some numerical simulations to verify the validity of theoretical analysis. It shows that the CNCS model is very valid for solving the 2D telegraph equations.

The remaining contents in this paper are scheduled as follows. In Sect. 2, we first review the spectral-collocation method and some Sobolev spaces. Then, in Sect. 3, we build the CNCS model for the 2D telegraph equations and analyze the existence, uniqueness, stability, and convergence of the CNCS solutions. Next, in Sect. 4, we use two sets of numerical examples to verify that the results of numerical computations are accorded with the theoretical analysis and to certify that the CNCS model is very valid for solving the 2D telegraph equations. Finally, we supply the main conclusions and discussion in Sect. 5.

## The CS method and some useful Sobolev spaces

### The CS method

Let $P_{N}$ be an interpolation subspace in a one- or two-dimensional space. The CS method consists in that the solution *u* of PDE is approximated with the interpolation polynomial $u_{N}$ in $P_{N}$, whose interpolation nodes adopt the so-called Chebyshev–Gauss–Lobatto (CGL) points (see [4]).

The Chebyshev polynomials are some special Jacobi polynomials, which are orthogonal with the Chebyshev weight function $\omega(x) = {1}/{\sqrt{1-x ^{2}}}$ over $[-1,1]$, namely,

$$ \int^{1}_{-1}T_{m}(x)T_{n}(x) \omega(x)\,\mathrm{d}x = \gamma_{n}\delta_{m,n}, $$

where

$$ \gamma_{n} = \Vert T_{n} \Vert ^{2}_{\omega}= \int ^{1}_{-1}T_{n}^{2}(x) \omega(x)\,\mathrm{d}x. $$

Let $\{x_{j}\}_{j=0}^{N}$ and $\{y_{k}\}_{k=0}^{N}$ be two sets of space nodes, that is, the CGL points in *x* and *y* directions, respectively, and let $\{\omega_{k}\}^{N}_{k=0}$ be a set of weights. They are, respectively, defined by

2 $$ x_{k} = -\cos\frac{\pi k}{N},\qquad y_{k} = -\cos\frac{k\pi}{N},\qquad\omega_{k} = \frac{\pi }{c_{k}N},\quad 0 \leq k\leq N, $$

where $c_{0} = c_{N} = 2$ and $c_{k} = 1$ ($k = 1,2,\ldots,N-1$). They have the following property (see, e.g., [3]).

#### Theorem 1

*Let* $\{x_{k}\}^{N}_{k = 0}$, $\{y_{k}\}_{k=0}^{N}$, *and* $\{\omega_{k} \}^{N}_{k=0}$ *be the sets of CGL quadrature nodes and weights*, *respectively*. *Then*

3 $$ \int^{1}_{-1} \int^{1}_{-1}p(x,y)\omega(x)\omega(y)\,\mathrm{d}x\,\mathrm{d}y= \sum^{N}_{j=0}\sum ^{N}_{k=0}p(x_{j},y_{k}) \omega_{j}\omega_{k},\quad\forall p(x,y)\in P_{2N-1}. $$

More specifically, the CS basic principle is to get an approximate solution for $u(x,y)$ by a sum

4 $$ u_{N}(x,y) = \sum_{j=0}^{N} \sum_{k=0}^{N}u_{N}(x_{j},y_{k})h_{j}(x)h _{k}(y), $$

where $u_{N}(x,y)\in P_{N}$, the interpolation nodes $\{x_{j}\}^{N} _{j=0}$ and $\{y_{k}\}^{N}_{k=0}$ are the CGL points given by (2), and $\{h_{j}(x)\}^{N}_{j=0}$ and $\{h_{k}(y)\}^{N}_{j=0}$ are the Lagrange basis polynomials associated with the sets of the CGL points $\{x_{j}\}^{N}_{j=0}$ and $\{y_{k}\}^{N}_{k=0}$, respectively.

Moreover, the derivative of $u_{N}(x,y)$ at $x_{k}$ is obtained by

5 $$ \frac{\partial u_{N}(x_{k},y)}{\partial x} = \sum^{N}_{j=0} \sum^{N} _{l=0}u_{N}(x_{j},y_{l})h^{\prime}_{j}(x_{k})h_{l}(y), \quad0\leq k\leq N, $$

where the first-order derivative $h_{j}'(x_{k})$ at the CGL points can be computed by the following formulas:

6 $$ h_{j}^{\prime}(x_{k}) = \textstyle\begin{cases} -\frac{2N^{2}+1}{6},&k = j = 0, \\ \frac{c_{k}}{c_{j}}\frac{(-1)^{k+j}}{x_{k}-x_{j}},& k \neq j,0 \leq k,j \leq N, \\ -\frac{x_{k}}{2(1-x^{2}_{k})},& 1 \leq k = j \leq N-1, \\ \frac{2N^{2}+1}{6},& k = j = N, \end{cases} $$

where $c_{0} = c_{N} = 2$ and $c_{k} = 1$ ($k = 1,2,\ldots,N-1$). By replacing *x* in (5) and (6) with *y*, we easily obtain the computational formulas of ${\partial u_{N}(x,y_{k})}/{\partial y}$.

### Some useful Sobolev spaces

First, we supply several useful Sobolev spaces, whose detailed descriptions can be found in [17].

Let $\Omega\in\mathbb{R}^{2}$ be a bounded open domain with boundary *∂*Ω, and let $L^{2}(\Omega)$ denote the set of all square-integrable functions defined on Ω, equipped with inner product and norm

$$ (u,v)= \int_{\Omega}uv \,\mathrm{d}x\,\mathrm{d}y\quad\mbox{and}\quad \Vert u \Vert _{0} = \biggl( \int_{\Omega}\vert u \vert^{2}\,\mathrm{d}x\,\mathrm{d}y\biggr) ^{1/2},\quad\forall u, v\in L^{2}(\Omega). $$

For a nonnegative integer *m* and $\alpha=(\alpha_{1},\alpha_{2})$ (where $\alpha_{i}\ge0$ are integers, and $\vert\alpha\vert =\alpha_{1}+ \alpha_{2}$), define

$$ H^{m}(\Omega)= \bigl\{ u\in L^{2}(\Omega): D^{\alpha}u \in L^{2}( \Omega), 0\leq\vert\alpha\vert\leq m \bigr\} , $$

equipped with norm and seminorm

$$ \Vert u \Vert _{m} = \biggl( \sum_{0\leq\vert\alpha\vert \leq m} \bigl\Vert D^{\alpha}u \bigr\Vert ^{2} _{0} \biggr) ^{1/2} $$

and

$$ \vert u \vert_{m} = \biggl( \sum _{ \vert\alpha\vert = m} \bigl\Vert D^{\alpha}u \bigr\Vert ^{2}_{0} \biggr) ^{1/2}, $$

where $D^{\alpha}u=\frac{\partial^{\vert\alpha\vert }u}{\partial x^{\alpha _{1}}\partial y^{\alpha_{2}}}$. Set $H^{m}_{0}(\Omega) = \{u\in H^{m}({\Omega}): D^{\alpha}u(x)| _{\partial\Omega} = 0, \vert\alpha\vert < m \}$ and let $H^{-m}(\Omega)$ denote the dual space of $H_{0}^{m}( \Omega)$.

Further, let $\omega=:\omega(x,y)=\omega(x)\omega(y) = {1}/{\sqrt{(1-x ^{2})(1-y^{2})}}$, $\Omega= (-1,1)^{2}$, and let $L^{2}_{\omega}( \Omega)$ denote the set of all square-integrable functions defined on Ω, equipped with norm

$$ \Vert u \Vert _{0,\omega} = \biggl( \int_{\Omega}\vert u \vert^{2}\omega \,\mathrm{d}x\,\mathrm{d}y\biggr) ^{1/2}, $$

and let $H^{m}_{\omega}(\Omega):=\{u\in L^{2}_{\omega}(\Omega): D ^{\alpha}u\in L^{2}_{\omega}(\Omega),0\leq\vert\alpha\vert \leq m\}$ be the weighted Sobolev space on Ω with the CGL quadrature weight function, equipped with the norm

$$ \Vert u \Vert _{m,\omega} = \biggl( \sum_{0\leq\vert\alpha \vert \leq m} \bigl\Vert D^{\alpha}u \bigr\Vert ^{2}_{0,\omega} \biggr) ^{\frac{1}{2}}. $$

Furthermore, let $H_{0,\omega}^{1}(\Omega) = \{u\in H^{1}_{\omega}( \Omega): u| _{\partial\Omega} = 0\}$, $(\cdot,\cdot)_{\omega}$ denote the weighted inter product of $L^{2}_{\omega}(\Omega)=H^{0} _{\omega}(\Omega)$, and let $\Vert \cdot \Vert _{H^{l}(H^{m}_{\omega})}$ be the norm in the space

$$ H^{l} \bigl(0, T; H^{m}_{\omega}(\Omega) \bigr)\equiv \Biggl\{ v(t)\in H^{m}_{ \omega}(\Omega): \Vert v \Vert _{H^{l}(H^{m}_{\omega})}^{2}\equiv \int_{0} ^{T}\sum_{i=0}^{l} \biggl\Vert \frac{\mathrm{d}^{i}}{\,\mathrm{d}t^{i}}v(t) \biggr\Vert _{m, \omega}^{2} \,\mathrm{d}t< \infty \Biggr\} . $$

Next, define the $H_{\omega}^{1}$-orthogonal projection $R_{N}: H _{0,\omega}^{1}(\Omega)\rightarrow P_{N}$ such that, for any $u\in H_{0,\omega}^{1}(\Omega)$,

$$ \bigl(\nabla(R_{N}u - u),\nabla v \bigr)_{\omega} = 0,\quad \forall v\in P_{N}, $$

or, equivalently,

7 $$ u_{N}(x,y) = R_{N}u(x,y) = \sum _{j=0}^{N}\sum_{k=0}^{N}u_{N}(x_{j}, y _{k})h_{j}(x)h_{k}(y). $$

Therefore we can also approximate the unknown solution $u(x,y)$ with $R_{N}u(x,y)$. Further, $R_{N}$ has the following important property (see [4, Chapter III]).

#### Theorem 2

*For any* $u\in H^{q}_{\omega}(\Omega)$ *with* $q\geq1$, *we have*

$$ \Vert \nabla R_{N}u \Vert _{0,\omega}\le \Vert \nabla u \Vert _{0,\omega},\qquad \bigl\Vert \partial^{k}(R_{N}u-u) \bigr\Vert _{0,\omega} \le CN^{k-q},\quad0\leq k\leq q\leq N+1, $$

*where* *C* *is a general positive constant independent of* *N* *and* Δ*t* *and used subsequently*.

Finally, we provide several formulas used often in the following discussions.

1. *The Poincaré inequality*. There exist a constant $C_{p}$ such that
  $$ C_{p}\Vert u \Vert _{m} \leq\vert u \vert _{m} \le \Vert u \Vert _{m},\quad\forall u\in H^{m}_{0}( \Omega). $$
2. *The Hölder inequality*.
  $$ \int_{\Omega}\vert uv \vert \,\mathrm{d}x\,\mathrm{d}y\leq \biggl( \int_{\Omega}\vert u \vert^{2}\,\mathrm{d}x\,\mathrm{d}y\biggr) ^{\frac{1}{2}} \biggl( \int_{\Omega}\vert v \vert^{2}\,\mathrm{d}x\,\mathrm{d}y\biggr) ^{\frac{1}{2}},\quad\forall u, v\in L^{2}(\Omega). $$
3. *Green’s formula*.
  $$ \int_{\Omega}v\Delta u \,\mathrm{d}x\,\mathrm{d}y= - \int_{\Omega}\nabla u\cdot\nabla v \,\mathrm{d}x\,\mathrm{d}y+ \int_{\partial\Omega}v \frac{\partial u}{\partial\boldsymbol{n}} \,\mathrm{d}s,\quad\forall u\in H^{2}(\Omega), \forall v\in H^{1}(\Omega), $$
  where $\Delta u={\partial^{2}u}/{\partial{x^{2}}}+{\partial^{2}u}/{\partial{y^{2}}}$, $\nabla u= ({\partial u}/{\partial x},{\partial u}/{\partial y})$, and ***n*** is the unit outer normal vector on *∂*Ω.
4. *The Cauchy inequality*.
  $$ ab\leq\frac{\varepsilon a^{2}}{2} + \frac{b^{2}}{2\varepsilon },\quad\forall a\geq0, b\geq0, \varepsilon> 0. $$

## The CNCS method for the 2D telegraph equations

### The analysis of the existence, uniqueness, and stability of weak solutions for the 2D telegraph equations

Since by using transforms $x' = -1+{2(x-a)}/{(b-a)}$ and $y' = -1+ {2(y-c)}/{(d-c)}$ we can ensure $[a,b]\leftrightarrow[-1,1]$ and $[c,d]\leftrightarrow[-1,1]$, respectively, for convenience, we can assume that $a = c = -1$ and $b = d =1$ in the subsequent discussions. By using Green’s formula we can obtain the following weak form for the 2D telegraph equations (1).

#### Problem 3

*Find* $u\in H^{2}(0,T; H_{0,\omega}^{1}(\Omega))$ *such that*

8 $$ \textstyle\begin{cases} (u_{tt},v)_{\omega} + {\mu}(\nabla u,\nabla v)_{\omega} + \alpha(u _{t}, v)_{\omega}+\beta(u, v)_{\omega}= (f,v)_{\omega},\quad \forall v\in H_{0,\omega}^{1}(\Omega), \\ u(x,y,0)=H(x,y),\qquad u_{t}(x,y,0)=G(x,y),\quad (x,y)\in\Omega. \end{cases} $$

In the following, we employ the variational principle (see, e.g., [3, 4]), and the Hölder and Cauchy inequalities to analyze the existence, uniqueness, and stability of the weak solution for Problem 3. We have the following main conclusion.

#### Theorem 4

*If* $f\in L^{2}(0,T;L^{2}_{\omega}(\Omega))$, $G\in L^{2}_{\omega}( \Omega)$, *and* $H\in H^{1}_{\omega}(\Omega)$, *then there exists a unique generalized solution* $u\in H^{2}(0,T; H_{0,\omega}^{1}(\Omega ))$ *for the variational formulation* (8) *satisfying the following stability*:

9 $$\begin{aligned} \Vert u_{t} \Vert _{0,\omega}+\Vert u \Vert _{1,\omega}\le\tilde{C} \bigl(\Vert G \Vert _{0,\omega }+ \Vert H \Vert _{1,\omega}+\Vert f \Vert _{L^{2}(H^{-1}_{\omega })} \bigr), \end{aligned}$$

*where* $\tilde{C}=2\sqrt{\max\{1, \beta, {1}/{(2\alpha)}\}/\min \{{\mu}, \beta\}}$.

#### Proof

Because (8) is equivalent to (1) and the system of equations (1) has a generalized solution *u* of other form, just as obtained in [10], which is a solution in (8), it is only necessary to demonstrate the uniqueness. Thus, we only need to prove that equation (8) has only a zero solution when $f(x,y,t) =H(x,y)=G(x,y)= 0$.

Taking $v = u_{t}$ in the first formula of equation (8), we have

10 $$ \frac{\mathrm{d}\Vert u_{t} \Vert ^{2}_{0,\omega }}{2\,\mathrm{d}t}+{\mu}\frac{\mathrm{d}\Vert \nabla u \Vert ^{2}_{0,\omega}}{2\,\mathrm{d}t}+\alpha \Vert u_{t} \Vert ^{2}_{0,\omega}+\beta\frac{\mathrm{d} \Vert u \Vert ^{2}_{0,\omega}}{2\,\mathrm{d}t} = (f,u_{t})_{\omega}. $$

By integrating (10) from 0 to $t\in[0,T]$ and by the Hölder and Cauchy inequalities we obtain

11 $$\begin{aligned} &\Vert u_{t} \Vert _{0,\omega}^{2} +\mu \Vert \nabla u \Vert _{0,\omega}^{2} + 2 \alpha\int_{0}^{t}\Vert u_{t} \Vert _{0,\omega}^{2} \,\mathrm{d}t+\beta \Vert u \Vert ^{2}_{0, \omega} \\ &\quad=\Vert G \Vert _{0,\omega}^{2}+\Vert \nabla H \Vert _{0,\omega}^{2}+\beta \Vert H \Vert ^{2} _{0,\omega}+2 \int_{0}^{t}(f,u_{t})_{\omega} \,\mathrm{d}t \\ &\quad\le \Vert G \Vert _{0,\omega}^{2}+\Vert \nabla H \Vert _{0,\omega}^{2}+\beta \Vert H \Vert ^{2}_{0,\omega}+ \frac{1}{2\alpha} \int_{0}^{t}\Vert f \Vert ^{2}_{0,\omega} \,\mathrm{d}t+2 \alpha \int_{0}^{t}\Vert u_{t} \Vert ^{2}_{0,\omega} \,\mathrm{d}t. \end{aligned}$$

Therefore, when $f(x,y,t) =H(x,y)=G(x,y)= 0$, we obtain $\Vert u \Vert _{0, \omega} = \Vert \nabla u \Vert _{0,\omega} = 0$, which implies $u = 0$, that is, equation (8) has a unique weak solution $u\in H^{1}_{0, \omega}(\Omega)$. Further, from (11) we obtain (9). This completes the proof of Theorem 4. □

### The CNCS method for the 2D telegraph equations

#### The establishment of the CNCS model

To establish the CNCS model for the 2D telegraph equations, it is necessary to discretize $u_{tt}$ and $u_{t}$ by means of the second-order difference quotient and spatial variables by means of the CNCS method. For this purpose, let $\{x_{j}\}_{j=0}^{N}$ and $\{y_{k}\}_{k=0}^{N}$ be the space nodes in *x* and *y* directions, respectively, with

$$ x_{j} = -\cos\frac{j\pi}{N},\qquad y_{k} = -\cos \frac{k\pi}{N}, $$

where the positive integer *N* denotes the number of nodes in a certain direction. For integer $K>0$, let $\Delta t=T/K $ be the time step, that is, $K\Delta t = T$. We approximate $u(x,y,n\Delta t)$ with $u^{n}$, $u_{t}$ with ${(u^{n+1}-u^{n-1})}/{(2\Delta t)}$, $u_{tt}$ with ${(u^{n+1}-2u^{n}+u^{n-1})}/{\Delta t^{2}}$, and $u^{n}(x,y)$ with $u_{N}^{n}(x,y)$, namely,

$$ u^{n}(x,y) \approx u_{N}^{n}(x,y) = \sum _{j=0}^{N}\sum_{k=0}^{N}u_{N} ^{n}(x_{j},y_{k})h_{j}(x)h_{k}(y), \quad0\leq n\leq K. $$

Thus, we can establish the following CNCS model for the 2D telegraph equations.

##### Problem 5

*Find* $u_{N}^{n} \in U_{N} \equiv H_{0,\omega}^{1}(\Omega)\cap P _{N}$ *such that*

12 $$ \textstyle\begin{cases} (u^{n+1}_{N}-2u^{n}_{N}+u^{n-1}_{N},v_{N})_{\omega} +\frac{{\mu} \Delta t^{2}}{2}(\nabla u_{N}^{n+1}+\nabla u_{N}^{n-1}, \nabla v_{N})_{ \omega} \\ \qquad{}+\frac{\alpha\Delta t}{2} (u_{N}^{n+1}-u_{N}^{n-1},v_{N})_{\omega}+\frac{ \beta\Delta t^{2}}{2}(u_{N}^{n+1}+u_{N}^{n-1}, v_{N})_{\omega} \\ \quad= \Delta t^{2}(f(t_{n}),v_{N})_{\omega}, \quad \forall v_{N}\in U_{N},1\leq n\leq K-1, \\ u^{0}_{N}(x,y) =R_{N} G(x,y),\qquad u^{1}_{N}(x,y) =u_{N}^{0}+2\Delta tR _{N} H(x,y),\quad(x,y)\in\Omega, \end{cases} $$

*where* $f(t_{n})=f(x,y,t_{n})$.

#### The analysis of the existence, uniqueness, and stability of the CNCS solutions

We further employ the Lax–Milgram theorem (see, e.g., [3]) and the Hölder and Cauchy inequalities to analyze the existence, uniqueness, and stability for the CNCS solutions. We have the following main conclusion.

##### Theorem 6

*If* $f\in L^{2}(0,T;L^{2}_{\omega}(\Omega))$, $G\in H^{1}_{\omega}( \Omega)$, *and* $H\in H^{1}_{\omega}(\Omega)$, *then there exists a unique sequence of solutions* $u_{N}^{n}\in U_{N}$ $(n=1, 2, \ldots, K)$ *for the CNCS model* (12) *satisfying the following stability*:

13 $$\begin{aligned} \bigl\Vert u_{N}^{n} \bigr\Vert _{1,\omega}&\le \biggl( \frac{8+{\mu} C_{p}^{2}+\beta}{C _{p}^{2}\min\{{\mu},\beta\}} \biggr) ^{1/2} \bigl( \Vert \nabla H \Vert _{0,\omega}+ \Vert \nabla G \Vert _{0,\omega} \bigr) \\ &\quad{}+ \Biggl( \frac{\Delta t}{\alpha\min\{{\mu},\beta\}}\sum_{j=1}^{n} \bigl\Vert f(t_{j}) \bigr\Vert _{0,\omega}^{2} \Biggr) ^{1/2},\quad n=1, 2, \ldots, K. \end{aligned}$$

##### Proof

Set $A(u,v)=(u,v)_{\omega} +\frac{{\mu}\Delta t ^{2}}{2}(\nabla u,\nabla v)_{\omega}+\frac{\alpha\Delta t}{2} (u,v)_{ \omega}+\frac{\beta\Delta t^{2}}{2}(u, v)_{\omega}$ and $F(v)=\Delta t^{2}(f(t_{n}), v)_{\omega }+(2u^{n}_{N}-u^{n-1}_{N},v)_{\omega } -\frac{{\mu}\Delta t^{2}}{2}(\nabla u_{N}^{n-1},\nabla v)_{\omega }+\frac{\alpha\Delta t}{2} (u_{N}^{n-1},v)_{\omega}-\frac{\beta \Delta t^{2}}{2}(u_{N}^{n-1}, v)_{\omega}$. Then Problem 5 can be rewritten as the following:

##### Problem 7

*Find* $u_{N}^{n} \in U_{N} \equiv H_{0,\omega}^{1}(\Omega)\cap P _{N}$ *such that*

14 $$ \textstyle\begin{cases} A(u^{n+1}_{N},v_{N})_{\omega}= F(v_{N})_{\omega}, \quad \forall v_{N}\in U_{N}, 1\leq n\leq K-1, \\ u^{0}_{N}(x,y) =R_{N} G(x,y),\qquad u^{1}_{N}(x,y) =u_{N}^{0}+2\Delta tR _{N} H(x,y),\quad(x,y)\in\Omega. \end{cases} $$

It is obvious that $A(\cdot,\cdot)$ is a bounded and positive definite bilinear functional on $U_{N}$ and, for given $f(t_{n})$, $u^{n}_{N}$, and $u^{n-1}_{N}$, $F(\cdot)$ is a bounded linear functional on $U_{N}$. Thus, by the Lax–Milgram theorem (see, e.g., [3]) Problem 7 has a unique sequence of solutions $u_{N}^{n} \in U_{N}$ $(n=1, 2, \ldots, K)$.

By taking $v_{N} = u_{N}^{n+1}-u_{N}^{n-1}$ in the first equation of (14), with the Hölder and Cauchy inequalities, we have

15 $$\begin{aligned} & \bigl\Vert u_{N}^{n+1}-u_{N}^{n} \bigr\Vert _{0,\omega}^{2}- \bigl\Vert u_{N}^{n}-u_{N}^{n-1} \bigr\Vert _{0,\omega}^{2}+ \frac{{\mu}\Delta t^{2}}{2} \bigl( \bigl\Vert \nabla u_{N}^{n+1} \bigl\Vert _{0,\omega}^{2}- \bigr\Vert \nabla u_{N}^{n-1} \bigr\Vert _{0,\omega}^{2} \bigr) \\ &\qquad{} +\frac{\alpha\Delta t}{2} \bigl\Vert u_{N}^{n+1}-u_{N}^{n-1} \bigr\Vert _{0,\omega}^{2} + \frac{\beta\Delta t^{2}}{2} \bigl( \bigl\Vert u_{N}^{n+1} \bigr\Vert _{0,\omega}^{2}- \bigl\Vert u_{N}^{n-1} \bigr\Vert _{0,\omega}^{2} \bigr) \\ &\quad= \Delta t^{2} \bigl(f(t_{n}),u_{N}^{n+1}-u _{N}^{n-1} \bigr)_{0,\omega} \\ &\quad\le\frac{\Delta t^{3}}{2\alpha} \bigl\Vert f(t_{n}) \bigr\Vert _{0,\omega}^{2}+\frac{ \alpha\Delta t}{2} \bigl\Vert u_{N}^{n+1}-u_{N}^{n-1} \bigr\Vert ^{2}_{0,\omega}. \end{aligned}$$

From (15) we obtain

16 $$\begin{aligned} & \bigl\Vert u_{N}^{n+1}-u_{N}^{n} \bigr\Vert _{0,\omega}^{2}- \bigl\Vert u_{N}^{n}-u_{N}^{n-1} \bigr\Vert _{0,\omega}^{2} \\ &\qquad {}+ \frac{{\mu}\Delta t^{2}}{2} \bigl( \bigl\Vert \nabla u_{N}^{n+1} \bigr\Vert _{0,\omega}^{2}- \bigl\Vert \nabla u_{N}^{n-1} \bigr\Vert _{0,\omega}^{2} \bigr) + \frac{\beta\Delta t^{2}}{2} \bigl( \bigl\Vert u_{N}^{n+1} \bigr\Vert _{0,\omega}^{2}- \bigl\Vert u_{N}^{n-1} \bigr\Vert _{0,\omega}^{2} \bigr) \\ &\quad \le\frac{\Delta t^{3}}{2 \alpha} \bigl\Vert f(t_{n}) \bigr\Vert _{0,\omega}^{2}. \end{aligned}$$

Summing (16) from 1 to *n* and using the second formula of (14), we obtain

17 $$\begin{aligned} & \bigl\Vert u_{N}^{n+1}-u_{N}^{n} \bigr\Vert _{0,\omega}^{2}+ \frac{{\mu}\Delta t^{2}}{2} \bigl( \bigl\Vert \nabla u_{N}^{n+1} \bigr\Vert _{0,\omega} ^{2}+ \bigl\Vert \nabla u_{N}^{n} \bigr\Vert _{0,\omega}^{2} \bigr) + \frac{\beta\Delta t^{2}}{2} \bigl( \bigl\Vert u_{N}^{n+1} \bigr\Vert _{0,\omega}^{2}+ \bigl\Vert u_{N}^{n} \bigr\Vert _{0, \omega}^{2} \bigr) \\ & \quad\le \bigl\Vert u_{N}^{1}-u_{N}^{0} \bigr\Vert _{0,\omega}^{2}+ \frac{{\mu}\Delta t^{2}}{2} \bigl( \bigl\Vert \nabla u_{N}^{1} \bigr\Vert _{0,\omega} ^{2}+ \bigl\Vert \nabla u_{N}^{0} \bigr\Vert _{0,\omega}^{2} \bigr) \\ &\qquad{} + \frac{\beta\Delta t^{2}}{2} \bigl( \bigl\Vert u_{N}^{1} \bigr\Vert _{0,\omega}^{2}+ \bigl\Vert u_{N}^{0} \bigr\Vert _{0,\omega}^{2} \bigr) + \frac{\Delta t^{3}}{2\alpha} \sum _{j=1}^{n} \bigl\Vert f(t_{j}) \bigr\Vert _{0,\omega}^{2} \\ &\quad\le\frac{4\Delta t^{2}}{C_{p}^{2}}\Vert \nabla R_{N}H \Vert _{0,\omega}^{2}+ \frac{{\mu}\Delta t^{2}}{2} \bigl( \Vert \nabla R_{N}H \Vert _{0,\omega}^{2}+ \Vert \nabla R_{N}G \Vert _{0,\omega}^{2} \bigr) \\ & \qquad{}+ \frac{\beta\Delta t^{2}}{2C_{P}^{2}} \bigl( \Vert \nabla R_{N}H \Vert _{0, \omega}^{2}+\Vert \nabla R_{N}G \Vert _{0,\omega}^{2} \bigr) + \frac{\Delta t^{3}}{2\alpha}\sum _{j=1}^{n} \bigl\Vert f(t_{j}) \bigr\Vert _{0,\omega}^{2} \\ &\quad\le \biggl( \frac{4\Delta t^{2}}{C_{p}^{2}}+ \frac{{\mu }\Delta t^{2}}{2}+ \frac{\beta\Delta t^{2}}{2C_{P}^{2}} \biggr) \bigl( \Vert \nabla H \Vert _{0,\omega}^{2}+\Vert \nabla G \Vert _{0,\omega}^{2} \bigr) \\ &\qquad{}+ \frac{ \Delta t^{3}}{2\alpha}\sum_{j=1}^{n} \bigl\Vert f(t_{j}) \bigr\Vert _{0,\omega}^{2},\quad n=1, 2, \ldots, K-1. \end{aligned}$$

Thus from (17) we obtain $\Vert \nabla u_{N}^{n} \Vert _{\omega}=\Vert u_{N}^{n} \Vert _{\omega}=0$ ($n=1, \ldots, K$) when $H(x,y)=G(x,y) = f(x,y,t) = 0$, which implies $u_{N}^{n}=0$ ($n=1, 2, \ldots, K$). In other words, the CNCS model (14) has a unique series of solutions. From (17) we immediately attain (13). This completes the proof of Theorem 6.  □

#### The analysis of convergence of the CNCS solutions

In the following, we employ the CS method in Sect. 2 and the Hölder and Cauchy inequalities to analyze the convergence for the CNCS solutions of Problem 5. We have the following main conclusion.

##### Theorem 8

*Under the conditions of Theorem *6, *the errors between the solution for Problem *3 *and the series of solutions of Problem *5 *have the following estimates*:

18 $$\begin{aligned} \bigl\Vert u(t_{n})-u_{N}^{n} \bigr\Vert _{1,\omega} \le C \bigl(\Delta t^{2}+N^{-2} \bigr),\quad1\leq n\leq K, \end{aligned}$$

*where* *C* *is a general positive constant independent to* *N* *and* Δ*t*.

##### Proof

If $u_{t}$ is approximated with ${(u^{n+1}-u^{n-1})}/ {2\Delta t}$ and $u_{tt}$ is approximated with $(u^{n+1}- 2u^{n}+u ^{n-1})/{\Delta t^{2}}$, then we obtain the following semidiscretized formulation of equation (8) in time:

19 $$\begin{aligned} \textstyle\begin{cases} (u^{n+1}-2u^{n}+u^{n-1},v)_{\omega} +\frac{{\mu}\Delta t^{2}}{2}( \nabla u^{n+1}+\nabla u^{n-1},\nabla v)_{\omega}+\frac{\alpha\Delta t}{2} (u^{n+1}-u^{n-1},v)_{\omega} \\ \quad {} +\frac{\beta\Delta t^{2}}{2}(u^{n+1}+u^{n-1}, v)_{\omega}= \Delta t^{2}(f(t_{n}),v)_{\omega}, \quad \forall v\in U, 1\leq n\leq K-1, \\ u^{0}(x,y) =G(x,y),\qquad u^{1}(x,y) =H(x,y),\quad(x,y)\in\Omega, \end{cases}\displaystyle \end{aligned}$$

Let $e_{1}^{n} = u(t_{n})-u^{n}$, $e_{2}^{n} = u^{n}- R_{N}u^{n}$, and $e^{n}_{3} = R_{N}u^{n}-u_{N}^{n}$.

1. First, estimate $e_{1}^{n}$.
  At time $t=t_{n}$, by applying Taylor’s expansion to (8) and subtracting (19), taking $v=e_{1}^{n+1}-e_{1}^{n-1}$, using Green’s formula and the Hölder and Cauchy inequalities, we obtain
  20 $$\begin{aligned} & \bigl\Vert e_{1}^{n+1}-e_{1}^{n} \bigr\Vert _{0,\omega}^{2}- \bigl\Vert e_{1}^{n}-e_{1}^{n-1} \bigr\Vert _{0,\omega}^{2}+\frac{{\mu}\Delta t^{2}}{2} \bigl( \bigl\Vert \nabla e_{1}^{n+1} \bigr\Vert _{0,\omega}^{2}- \bigl\Vert \nabla e_{1}^{n-1} \bigr\Vert _{0,\omega}^{2} \bigr) \\ &\qquad{} +\alpha\Delta t \bigl\Vert e_{1}^{n+1}-e_{1}^{n-1} \bigr\Vert _{0,\omega}^{2}+\beta\Delta t^{2} \bigl( \bigl\Vert e_{1}^{n+1} \bigr\Vert _{0,\omega}^{2}- \bigl\Vert e_{1}^{n-1} \bigr\Vert _{0, \omega}^{2} \bigr) \\ &\quad= \frac{\Delta t^{4}}{12} \bigl(u_{tttt} \bigl({\xi_{1}^{n}} \bigr),e_{1}^{n+1}-e_{1} ^{n-1} \bigr)_{\omega} -\frac{{\mu}\Delta t^{4}}{2} \bigl(\Delta u_{tt} \bigl({\xi _{2}^{n}} \bigr),e_{1}^{n+1}-e_{1}^{n-1} \bigr)_{\omega} \\ & \qquad{}+\frac{\alpha\Delta t^{4}}{6} \bigl(u_{ttt} \bigl({ \xi_{3}^{n}} \bigr),e_{1}^{n+1}-e _{1}^{n} \bigr)_{\omega} +\frac{\beta\Delta t^{4}}{2} \bigl(u_{tt} \bigl({ \xi_{2}^{n}} \bigr),e _{1}^{n+1}-e_{1}^{n-1} \bigr)_{\omega} \\ & \quad\le\alpha\Delta t \bigl\Vert e_{1}^{n+1}-e_{1}^{n-1} \bigr\Vert _{0,\omega}^{2}+\frac{ \Delta t^{7}}{144\alpha} \bigl\Vert u_{tttt} \bigl({\xi_{1}^{n}} \bigr) \bigr\Vert _{0,\omega}^{2}+ \frac{{\mu}^{2}\Delta t^{7}}{4\alpha} \bigl\Vert \Delta u_{tt} \bigl({\xi_{2}^{n}} \bigr) \bigr\Vert _{0,\omega}^{2} \\ & \qquad{}+\frac{\alpha\Delta t^{7}}{36} \bigl\Vert u_{ttt} \bigl({ \xi_{3}^{n}} \bigr) \bigr\Vert _{0,\omega} ^{2}+\frac{\beta^{2}\Delta t^{7}}{4\alpha} \bigl\Vert u_{tt} \bigl({ \xi_{2}^{n}} \bigr) \bigr\Vert _{0,\omega}^{2}, \end{aligned}$$
  where $t_{n}\leq{\xi_{1}^{n}}$, ${\xi_{2}^{n}}$, ${\xi_{3}^{n}} \leq t _{n+1}$. Because $e_{1}^{1}=e_{1}^{0}=0$, simplifying and summing (20) from 1 to *n*, we obtain
  21 $$\begin{aligned} & 2 \bigl\Vert e_{1}^{n+1}-e_{1}^{n} \bigr\Vert _{0,\omega}^{2}+{\Delta t^{2}} \bigl( \bigl\Vert \nabla e_{1}^{n+1} \bigr\Vert _{0,\omega}^{2}+ \bigl\Vert \nabla e_{1}^{n} \bigr\Vert _{0,\omega}^{2} \bigr) +2 \beta\Delta t^{2} \bigl( \bigl\Vert e_{1}^{n+1} \bigr\Vert _{0,\omega}^{2}+ \bigl\Vert e_{1}^{n} \bigr\Vert _{0,\omega}^{2} \bigr) \\ & \quad\leq{C^{2}(u)} {\min\{{\mu},2\beta\}}\Delta t^{6}, \end{aligned}$$
  where
  $$\begin{aligned} C^{2}(u)&=\frac{1}{72\alpha\min\{{\mu},2\beta\}} \bigl[ \bigl\Vert u_{tttt}\bigl({\xi_{1}^{n}}\bigr) \bigr\Vert _{0,\omega}^{2} +18{\mu}^{2} \bigl\Vert \Delta u_{tt}\bigl( {\xi_{2}^{n}}\bigr)\bigr\Vert _{0,\omega}^{2} \\ &\quad {}+4\alpha^{2} \bigl\Vert u_{ttt}\bigl({\xi_{3}^{n}}\bigr) \bigr\Vert _{0,\omega}^{2} + 36\beta^{2} \bigl\Vert u_{tt}\bigl({\xi_{2}^{n}}\bigr) \bigr\Vert _{0,\omega}^{2}\bigr]. \end{aligned}$$
  Further, we obtain
  22 $$ \bigl\Vert e_{1}^{n} \bigr\Vert _{1,\omega}\leq C(u)\Delta t^{2}. $$
2. Next, estimate $e_{2}$.
  The estimate of $e_{2}$ can be immediately obtained by Theorem 2, that is, when $u^{n}\in H^{3}(\Omega)$,
  23 $$\begin{aligned} & \bigl\Vert e_{2}^{n} \bigr\Vert _{1,\omega} \le CN^{-2},\quad n=1, 2, \ldots, K. \end{aligned}$$
3. Finally, estimate $e_{3}=R_{N}u^{n}-u_{N}^{n}$.
  Subtracting Problem 5 from (19) taking $v=v_{N}\in U_{N}$, we obtain
  24 $$\begin{aligned} & \bigl(u^{n+1}-u_{N}^{n+1}-2 \bigl(u^{n}-u_{N}^{n} \bigr)+u^{n-1}-u_{N}^{n-1},v_{N} \bigr)_{ \omega} \\ &\quad\quad {}+\frac{{\mu}\Delta t^{2}}{2} \bigl(\nabla \bigl(u^{n+1}-u_{N}^{n+1} \bigr)+\nabla \bigl(u ^{n-1}-u_{N}^{n-1} \bigr), \nabla v_{N} \bigr)_{\omega} \\ &\quad\quad {} +\frac{\alpha\Delta t}{2} \bigl(u^{n+1}-u_{N}^{n+1}- \bigl(u^{n-1}-u_{N}^{n-1} \bigr),v _{N} \bigr)_{\omega} \\ &\quad\quad {}+\frac{\beta\Delta t^{2}}{2} \bigl(u^{n+1}-u_{N}^{n+1}+u^{n-1}-u_{N}^{n-1}, v_{N} \bigr)_{\omega} \\ &\quad =0,\quad\forall v_{N}\in U_{N}, \end{aligned}$$
  By Theorem 2, (24), the property of $R_{N}$, the Hölder and Cauchy inequalities, and Taylor’s formula we have
  25 $$\begin{aligned} & \bigl\Vert e_{3}^{n+1}-e_{3}^{n} \bigr\Vert _{0,\omega}^{2}- \bigl\Vert e_{3}^{n}-e_{3}^{n-1} \bigr\Vert _{0,\omega}^{2}+\frac{{\mu}\Delta t^{2}}{2} \bigl( \bigl\Vert \nabla e_{3}^{n+1} \bigr\Vert _{0,\omega}^{2}- \bigl\Vert \nabla e_{3}^{n-1} \bigr\Vert _{0,\omega}^{2} \bigr) \\ &\qquad{}+\frac{\alpha\Delta t}{2} \bigl\Vert e_{3}^{n+1}-e_{3}^{n-1} \bigr\Vert _{0,\omega}^{2}+ \beta\Delta t^{2} \bigl( \bigl\Vert e_{3}^{n+1} \bigr\Vert _{0,\omega}^{2}- \bigl\Vert e_{3}^{n-1} \bigr\Vert _{0,\omega}^{2} \bigr) \\ &\quad= \bigl(u^{n+1}-2u^{n}+u^{n-1}- \bigl(u_{N}^{n+1}-2u_{N}^{n}+u_{N}^{n-1} \bigr),e_{3} ^{n+1}-e_{3}^{n-1} \bigr)_{\omega} \\ &\qquad{}+ \bigl(R_{N}u^{n+1}-u^{n+1}-2 \bigl(R_{N}u^{n}-u^{n} \bigr)+ \bigl(R_{N}u^{n-1}-u^{n-1} \bigr),e _{3}^{n+1}-e_{3}^{n-1} \bigr)_{\omega} \\ &\qquad{} +\frac{{\mu}\Delta t^{2}}{2} \bigl(\nabla \bigl(u^{n+1}-u_{N}^{n+1} \bigr)+\nabla \bigl(u ^{n-1}-u_{N}^{n-1} \bigr), \nabla \bigl(e_{3}^{n+1}-e_{3}^{n-1} \bigr) \bigr)_{\omega} \\ &\qquad{} +\frac{{\mu}\Delta t^{2}}{2} \bigl(\nabla \bigl(R_{N}u^{n+1}-u^{n+1} \bigr)+\nabla \bigl(R _{N}u^{n-1}-u^{n-1} \bigr), \nabla \bigl(e_{3}^{n+1}-e_{3}^{n-1} \bigr) \bigr)_{\omega} \\ &\qquad{} +\frac{\beta\Delta t^{2}}{2} \bigl(u^{n+1}-u_{N}^{n+1}+u^{n-1}-u_{N}^{n-1}, e_{3}^{n+1}-e_{3}^{n-1} \bigr)_{\omega} \\ &\qquad{} +\frac{\beta\Delta t^{2}}{2} \bigl(R_{N}u^{n+1}-u^{n+1}+R_{N}u^{n-1}-u^{n-1}, e_{3}^{n+1}-e_{3}^{n-1} \bigr)_{\omega} \\ & \quad= \bigl(R_{N}u^{n+1}-u^{n+1}-2 \bigl(R_{N}u^{n}-u^{n} \bigr)+ \bigl(R_{N}u^{n-1}-u^{n-1} \bigr),e _{3}^{n+1}-e_{3}^{n-1} \bigr)_{\omega} \\ &\qquad{}+\frac{\beta\Delta t^{2}}{2} \bigl(R_{N}u^{n+1}-u^{n+1}+R_{N}u^{n-1}-u^{n-1}, e_{3}^{n+1}-e_{3}^{n-1} \bigr)_{\omega} \\ &\quad\le\frac{\alpha\Delta t}{2} \bigl\Vert e_{3}^{n+1}-e_{3}^{n-1} \bigr\Vert _{0,\omega} ^{2}+C\Delta t^{3}N^{-4}, \quad n=0, 1, \ldots, K-1. \end{aligned}$$
  Because $e_{3}^{1}=e_{3}^{0}=0$, summing (25) from 1 to *n*, we get
  26 $$\begin{aligned} & \bigl\Vert e_{3}^{n+1}-e_{3}^{n} \bigr\Vert _{0,\omega}^{2}+ \frac{{\mu}\Delta t^{2}}{2} \bigl( \bigl\Vert \nabla e_{3}^{n+1} \bigr\Vert _{0,\omega}^{2}+ \bigl\Vert \nabla e_{3}^{n} \bigr\Vert _{0,\omega}^{2} \bigr) +\beta\Delta t^{2} \bigl( \bigl\Vert e_{3}^{n+1} \bigr\Vert _{0,\omega}^{2}+ \bigl\Vert e_{3}^{n} \bigr\Vert _{0,\omega}^{2} \bigr) \\ &\quad\le C\Delta t^{2}N^{-4},\quad n=1,2, \ldots, K. \end{aligned}$$
  Thus we obtain
  27 $$ \bigl\Vert e_{3}^{n} \bigr\Vert _{1,\omega}\le CN^{-2},\quad n=1,2, \ldots, K. $$
  By combining (22)–(23) with (27) we get (18). This completes the proof of Theorem 8.

 □

##### Remark 1

Theorems 6 shows that in the CNCS model, that is, Problem 5, for the 2D telegraph equations, there exists a unique series of the solutions that is stable and continuously depends on the initial value and source functions. In order that the error estimates in Theorem 8 attain an optimal order, it is necessary to take the time-step Δ*t* and *N* satisfying $\Delta t\simeq N ^{-1}$. This theoretically ensures that Problem 5 is effective and reliable for solving the 2D telegraph equations.

## Numerical experiments

In this section, we utilize two sets of numerical experiments to verify the correction of the theoretical results of the CNCS model, that is, Problem 5, for the 2D telegraph equations. These numerical examples are implemented by Matlab software in Laptop (Microsoft Surface Book: Int Core i7 Processor, 16 GB RAM).

### Example 1

In the 2D telegraph equation (1), we take $\bar{\Omega }=[-1,1]\times[-1, 1]$; $L=\hat{C}=R=G=1$, that is, $\alpha=1$, $\beta= 2$, ${\mu}=1$; $\varphi(\pm1,y,t)=(1-\cos2\pi y)\exp(-t)$ ($-1\le y\le1$ and $t\in[0, \infty)$), $\varphi(x,\pm1,t)=(1- \cos2\pi x)\exp(-t)$ ($-1\le x\le1$ and $t\in[0, \infty)$), $H(x,y) = 1-\cos2\pi x\cos2\pi y$, $G(x,y) = \cos2\pi x\cos2 \pi y-1$, and $f(x,y,t) =3\exp(-t) + (8\pi^{2}-3)\cos2\pi x\cos2 \pi y\exp(-t)$. Thus we can find the following analytical solutions for the telegraph equations (1):

$$\begin{aligned} u(x,y,t)=(1-\cos2\pi x\cos2\pi y)\exp(-t),\quad(x,y,t)\in[-1, 1]\times[-1, 1] \times(0, \infty). \end{aligned}$$

When we take the time step $\Delta t = 0.01$ and the number of nodes in every direction $N = 100$, from Theorem 8, the theoretical errors between the analytical solution and the CNCS solutions $u_{N}^{n}$ $(n=1, 2, \ldots, K)$ should be $O(10^{-4})$.

**Figure 1:**
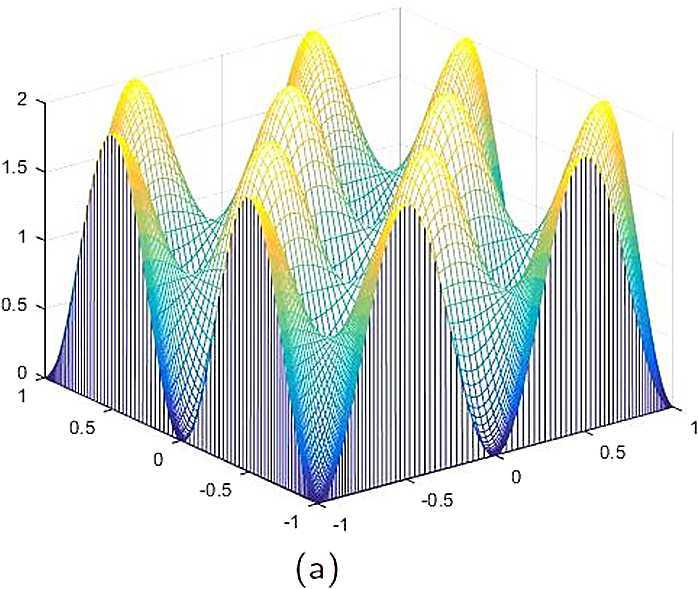

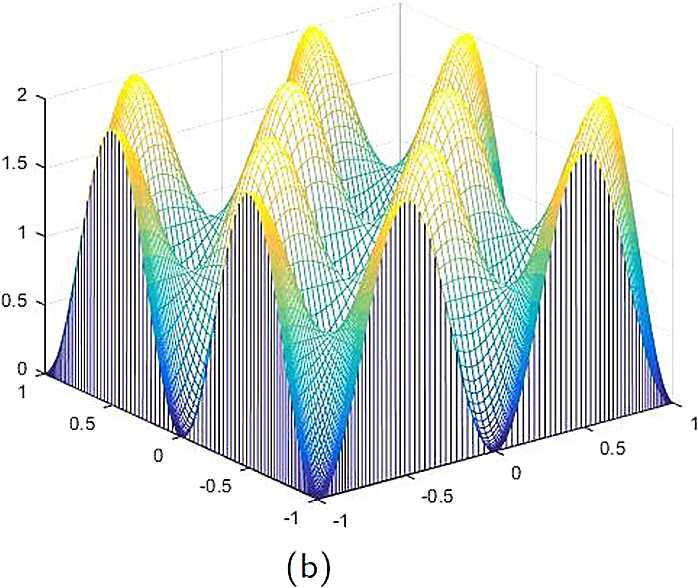

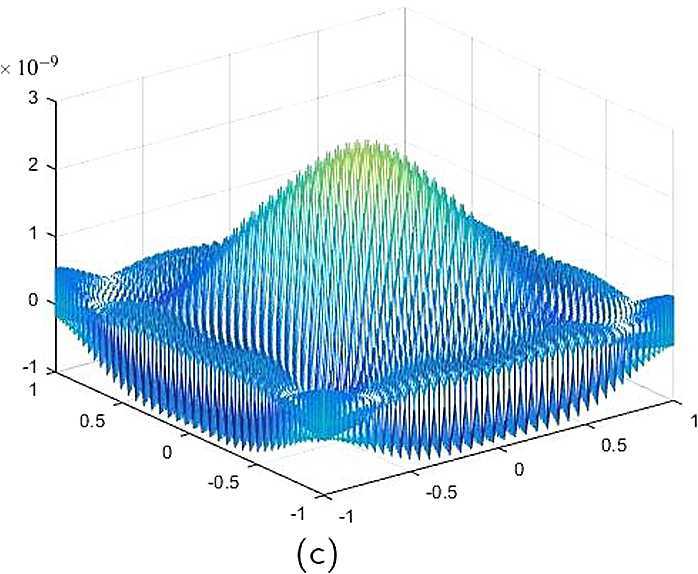
**(a)** The CNCS solution when $t=0.0$. **(b)** The analytical solution when $t=0.0$. **(c)** The errors between the analytical solution and CNCS solution at $t=0.0$

**Figure 2:**
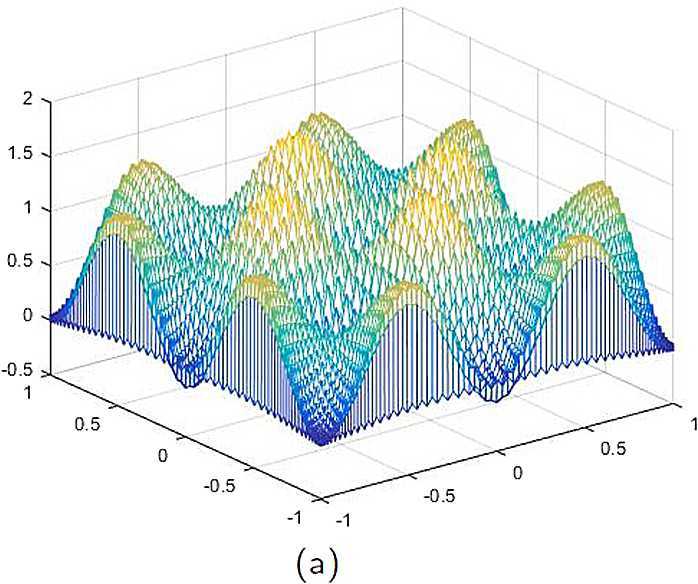

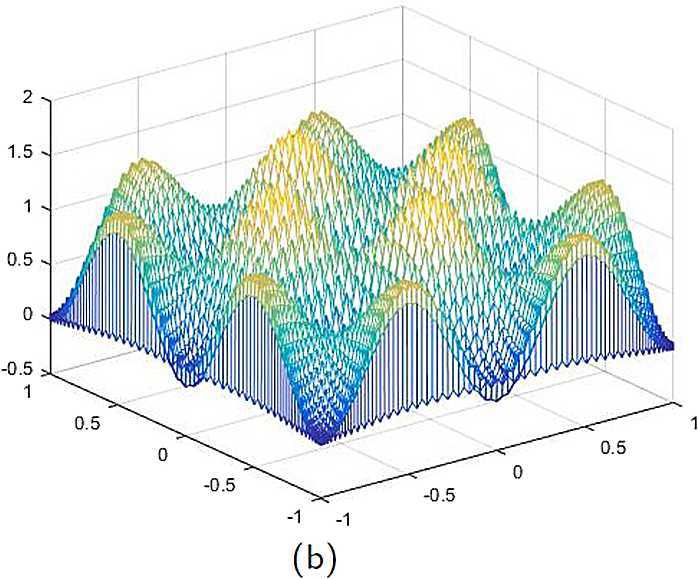

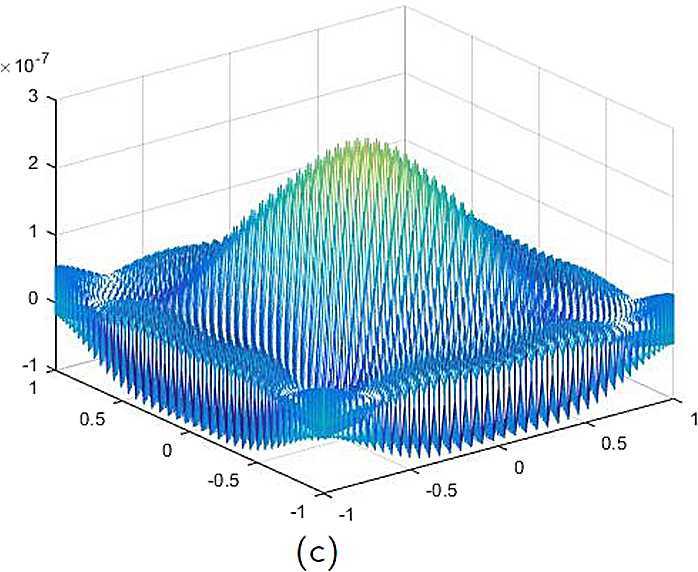
**(a)** The CNCS solution when $t=0.3$. **(b)** The analytical solution when $t=0.3$. **(c)** The errors between the analytical solution and CNCS solution at $t=0.3$

**Figure 3:**
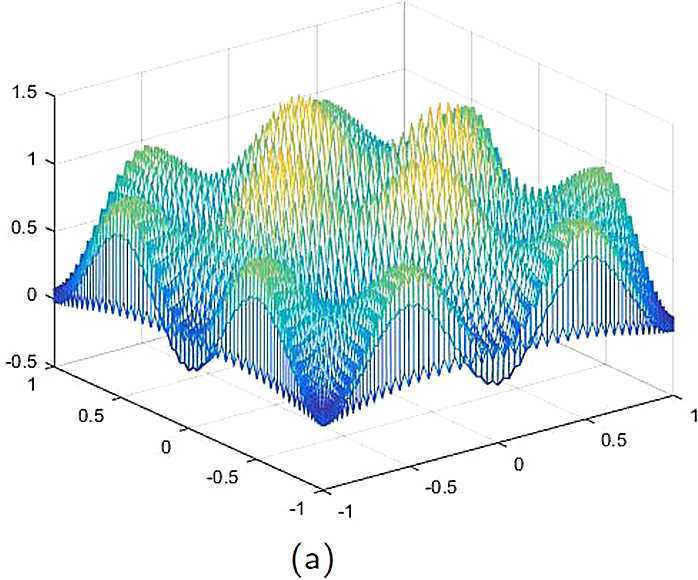

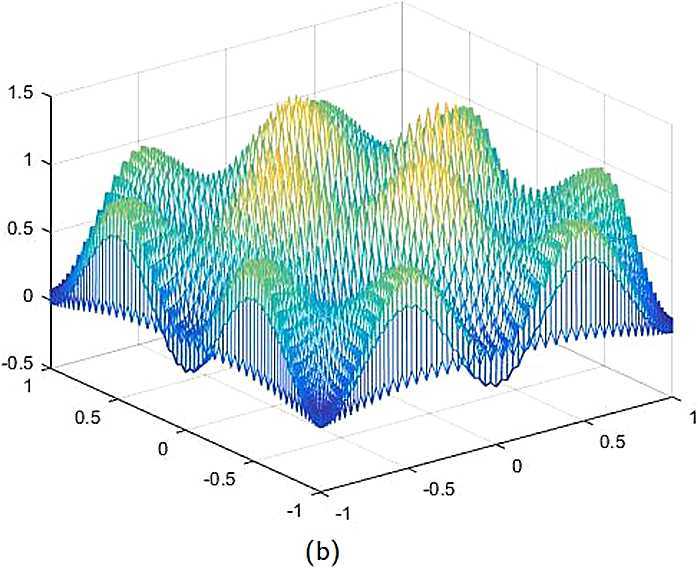

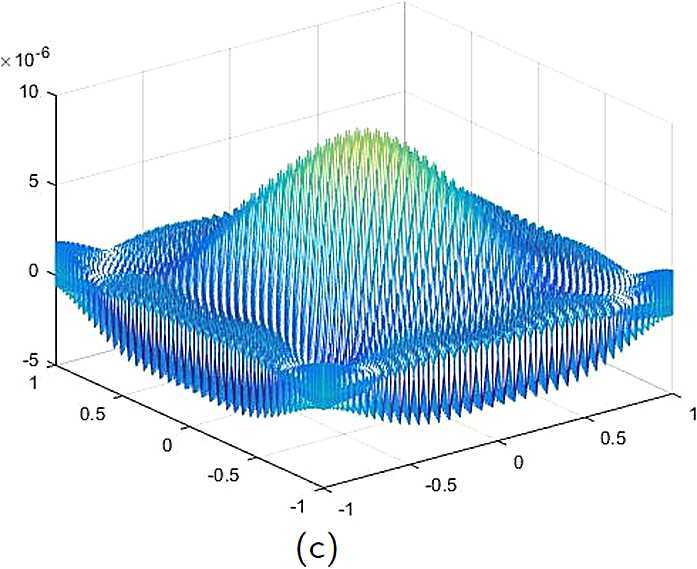
**(a)** The CNCS solution when $t=0.5$. **(b)** The analytical solution when $t=0.5$. **(c)** The errors between the analytical solution and CNCS solution at $t=0.5$

**Figure 4:**
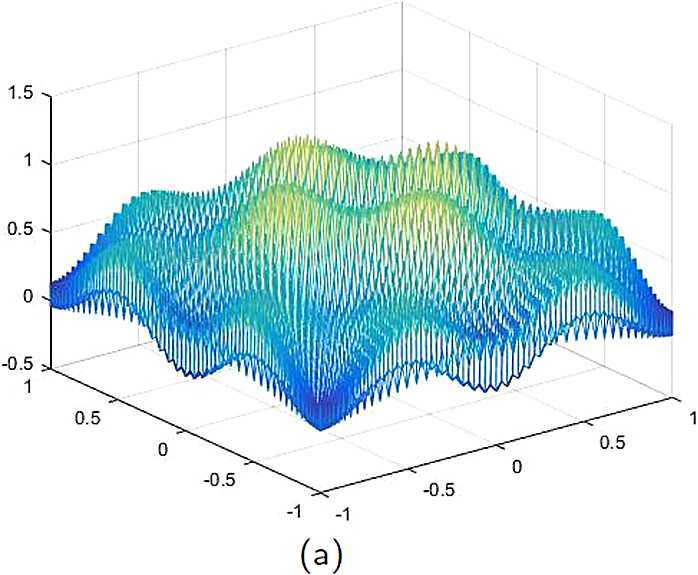

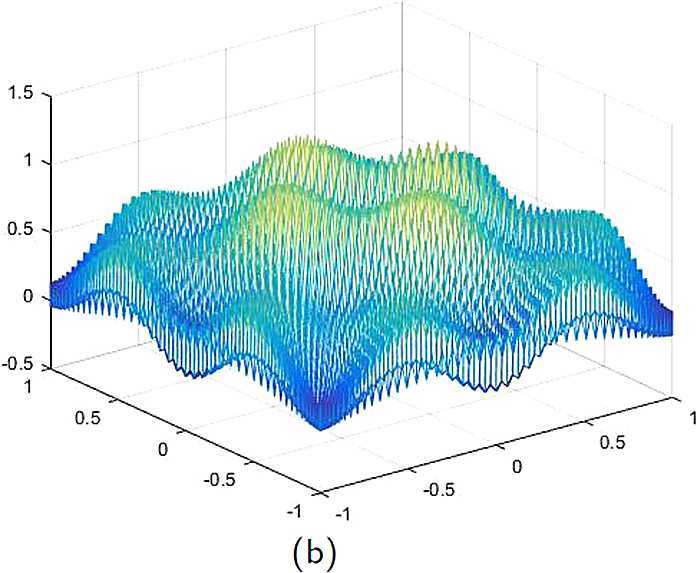

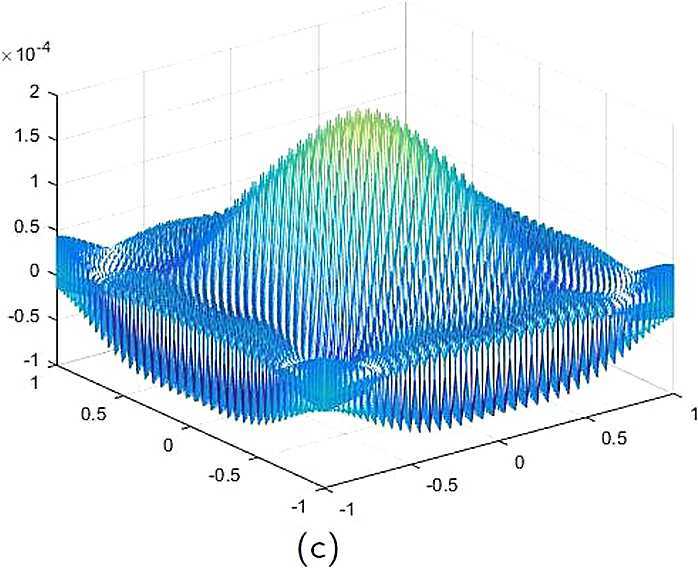
**(a)** The CNCS solution when $t=0.9$. **(b)** The analytical solution when $t=0.9$. **(c)** The errors between the analytical solution and CNCS solution at $t=0.9$

By the CNCS model, Problem 5, we can compute out the CNCS solutions at $T =0.0$, 0.3, 0.5, 0.9, depicted in photos (a) in Figs. 1–4, respectively. The analytical solutions at the same time nodes are depicted in photos (b) in Figs. 1–4, respectively. The errors between analytical solutions and the CNCS solutions at $t=0.0$, 0.3, 0.5, 0.9 are depicted in photos (c) of Figs. 1–4, respectively. Photos (a) and (b) in Figs. 1–4 are almost the same, which indicates that the numerical computational errors are accorded with the theoretical ones, because both errors are not greater than $O(10^{-4})$. This implies that the CNCS model is efficient and feasible for solving the 2D telegraph equations.

### Example 2

In the 2D telegraph equation (1), we still took $\bar{\Omega }=[-1, 1]\times[-1, 1]$; $L=\hat{C}=R=G=1$, that is, $\alpha=1$, $\beta= 2$, ${\mu}=1$; $\varphi(\pm1,y,t)=0$ ($-1\le y\le1$ and $t\in[0, \infty)$), $\varphi(x,\pm1,t)=-\sin\pi x\exp(0.5t)$ ($-1\le x\le1$ and $t\in[0, \infty)$), $H(x,y) = \sin\pi x\cos \pi y$, $G(x,y) = 0.5\sin\pi x \cos\pi y$, and $f(x,y,t) = (2.75+2\pi ^{2})\sin\pi x\cos\pi y\exp(0.5t)$. Thus we can find the following analytical solutions for the telegraph equations (1):

$$\begin{aligned} u(x,y,t)=\sin\pi x\cos\pi y\exp(0.5t),\quad(x,y,t)\in[-1, 1]\times [-1, 1] \times(0, \infty). \end{aligned}$$

When we take the time step $\Delta t = 0.01$ and the number of nodes in every direction $N = 100$, by Theorem 8 the theoretical errors between the analytical solution and the CNCS solutions $u_{N}^{n}$ $(n=1, 2, \ldots, K)$ still is $O(10^{-4})$.

**Figure 5:**
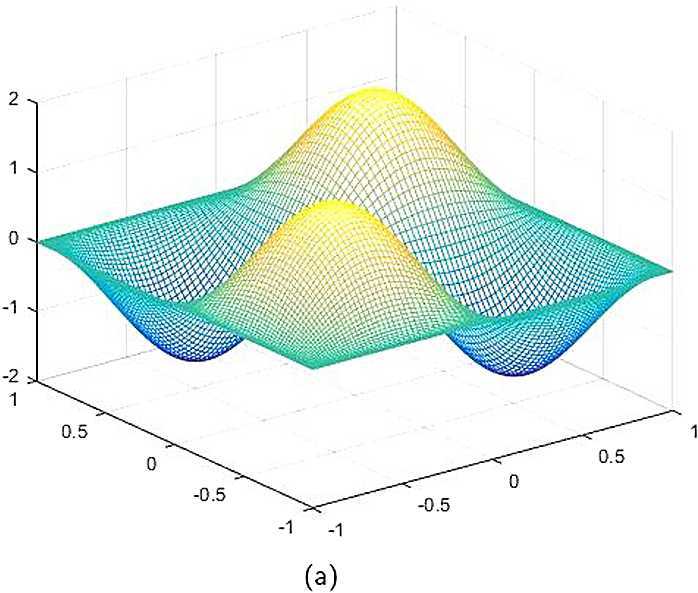

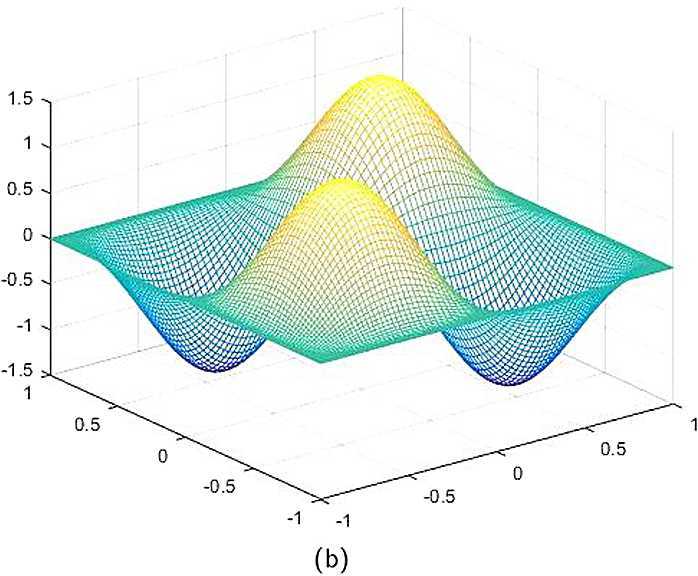

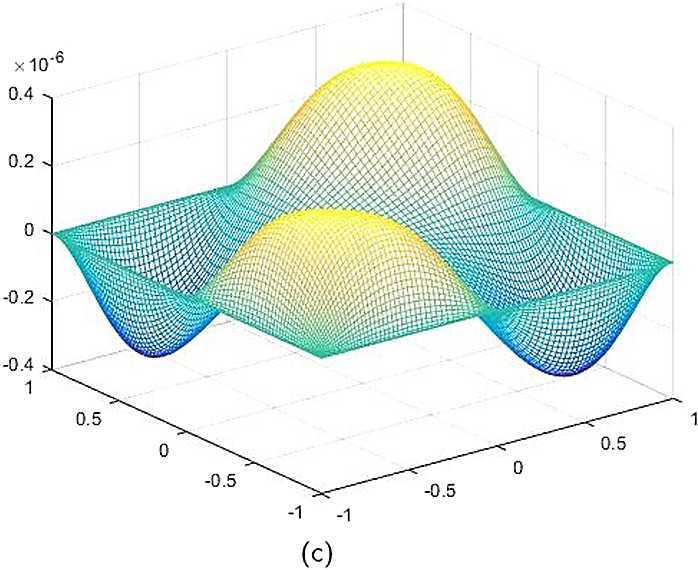
**(a)** The CNCS solution when $t=0.9$. **(b)** The analytical solution when $t=0.9$. **(c)** The errors between the analytical solution and CNCS solution at $t=0.9$

With the CNCS model, Problem 5, we compute out the CNCS solution at $T =0.9$ and depict it in photo (a) of Fig. 5. The analytical solutions at the same time node is depicted in photo (b) of Fig. 5. We also compute out the the error between the analytical solution and CNCS solution at $T =0.9$ and depict it in photo (c), which shows that the numerical errors are not greater than $O(10^{-4})$. This further explains that the CNCS model, Problem 5, is efficient and feasible for finding the numerical solutions of the 2D telegraph equations.

#### Remark 2

The accuracy of the CNCS solutions is far higher than other numerical methods, for example, the time–space FEM. For instance, in [10], though the time step is taken as 0.0025 and the space step is taken as 0.0000625, the accuracy of the time–space FEM solutions only attains 10^−3^, whereas our time-step is only 0.01 and $N=100$, or, equivalently, the space step is also taken as 0.01, but the accuracy of the CNCS solutions can attain 10^−6^.

## Conclusions and discussion

In this work, we have established the CNCS model by means of the Chebyshev polynomials for the 2D telegraph equations and discussed the existence, uniqueness, stability, and convergence of the CNCS solutions. We have also used two sets of numerical experiments to check the feasibility and effectiveness of the CNCS model and to verity that the numerical computing consequences accord with the theoretical ones. Moreover, we have shown that the CNCS model is very valid for solving the 2D telegraph equations.

Even if we only study the CNCS method for the 2D telegraph equations, the CNCS method can be easily and effectively used to solve for the telegraph equations in the three-dimensional space or the telegraph equations with complex geometric domains.

However, just as mentioned in [8] and [9], to ensure the CNCS solutions to attain the sufficiently high accuracy, we need to choose large *N*. Thus it causes large errors in the elements of the matrix, and the rounding errors are accumulated very quickly in the numerical computations. It is necessary to settle the key issue. We intend to use a proper orthogonal decomposition to reduce the CNCS model into the reduced-order CNCS format with very few unknowns in other paper, so that it can greatly lessen the accumulation of the rounding errors.
